# On the Measurements and Discrepancies Between Declared and Actual Thermal Performance of Reflective Insulation

**DOI:** 10.3390/ma19143060

**Published:** 2026-07-16

**Authors:** Tomasz Kisilewicz

**Affiliations:** Faculty of Civil Engineering, Cracow University of Technology, Warszawska Str. 24, 31-155 Kraków, Poland; tomasz.kisilewicz@pk.edu.pl

**Keywords:** reflective insulation, thermal resistance, infrared radiation, emissivity

## Abstract

**Highlights:**

- Marketing claims of infrared reflective performance should be independently verified.
- Surface visible appearance alone is not a reliable indicator of infrared reflective performance.
- Thermal imaging can be used to quantitatively assess infrared emissivity.
- Controlled laboratory testing offers a simple and effective approach to evaluating reflective insulation systems.
- Any coating applied to a reflective aluminum surface can substantially diminish its reflective effectiveness.

**Abstract:**

Infrared reflective materials are increasingly used in building applications; however, their actual reflective performance is not always consistent with manufacturers’ claims. Reliable and accessible methods for evaluating their thermal and reflective properties are therefore needed. This study aimed to develop and validate a practical laboratory procedure for assessing the reflective properties of building materials. The proposed method combines comparative reflectance testing with measurements of thermal resistance. The procedure employs commonly available laboratory equipment, including a high-resolution thermal imaging camera and a plate apparatus used for thermal conductivity evaluation. Five commercially available materials with an aluminum reflective layer were tested using this method. The laboratory measurements showed good agreement with ISO standard-based calculations, confirming the reliability of the method. Among the five tested materials, only two exhibited the declared reflective properties. Scanning electron reflectometry analysis revealed that poor-performing materials either lacked aluminum particles in the surface layer or contained aluminum coatings that were too thin. The proposed procedure provides a simple and effective method for evaluating reflective insulation materials. The results demonstrate that not all products marketed as reflective insulation possess the required reflective characteristics, and that aluminum layer thickness and surface composition are critical factors affecting thermal performance.

## 1. Introduction

The idea of reducing heat transfer through effective reflection or low emission of long-wave radiation has been known and used for a long time in many countries around the world. Currently used solutions typically involve multi-layer combinations of highly reflective materials, porous materials with low thermal conductivity, and air gaps.

There is a group of materials also referred to as reflective that concern building protection against excessive absorption of solar radiation. In this case, reflection of the shortwave solar radiation is crucial. This paper covers only far infrared reflective materials that significantly decrease heat transfer through the building shell.

The literature on this topic is extensive and covers many different research and practical aspects. Below, the main literature references showing new solutions in this field, verification of reflective insulation performance, methods of emissivity and thermal resistance testing and comparisons with standard insulation are briefly presented.

Lee et al. [1] conducted a broad review of reflective insulation (RI) systems based on 105 bibliographic items. It focused on radiant barrier and reflective insulation testing and calculation methods. Detailed analysis was devoted to possible degradation of surface emissivity due to possible water vapor condensation, dust buildup and metal oxidation. The authors pointed out that measurements using local sensors may overestimate thermal resistance without taking into account the varying convection intensity distribution. Iharaa et al. [2] also considered accelerated aging of treated aluminum used for façade cladding. They observed that the change in the reflectance of the tested samples in the infrared range may be due to accumulation of soil/dust and not due to surface aging.

Ashhar et al. [3] reviewed 91 research papers on the performance of reflective foils as thermal roof insulation, covering all the work done from 2000 to 2021. Research has shown that weather and climatic conditions have a huge impact on the effectiveness of reflective foil. RI may be very effective insulation in one location but ineffective in another location with a different type of weather. Fantucci et al. [4] were looking for a solution that can be used to conduct attic retrofitting interventions, with the aim of achieving better thermal performance in the summer period. The applied system enabled up to a 53% reduction of the summer heat gains.

Bruno et al. [5] performed experiments in climate chambers and simulated multilayer systems made of insulating panels. The authors claim that the adopted simulation infrared emissivity values of the tested materials were based on the manufacturer’s datasheet and verified by infrared camera measurements. Due to the reflective panel installed at the middle of the air–gap, thermal resistance was increased by approximately 7 times. The work [6] addressed the combined effect of temperature difference, long-wave atmospheric heat exchanges and solar radiation on the thermal resistance of walls with radiative insulation.

The authors of [7] focused on systems composed of multiple (up to 13) thin layers of various materials and air gaps. In the selected variants, the material surface was covered with a 16 μm-thick aluminum foil and emissivity equal to 0.04. The results of these studies indicate that it was possible to achieve up to a 72% reduction in the amount of conventional material, as well as a reduction in cost and embodied energy compared to a uniform insulating layer.

Zhou et al. [8] investigated a high-temperature multilayer insulation that consisted of reflective aluminum screens separated by thin (2 mm) layers of fibrous insulation. Aluminum tape with emissivity equal to 0.2 was used as a reflective screen. In this very specific case thermal insulation performance loaded with 10 layers of reflective screens was almost the same as with 5 layers. Therefore, the authors suggested that it is not advisable to increase the number of reflection screens to reduce heat transfer.

Kosny et al. [9] discussed the development, testing, and thermal performance of a radiant barrier containing a 1 cm-thick aerogel insulation core. The aerogel-based radiant barrier was tested in laboratory and deployed in a side-by-side field test with conventional insulation technology.

Lazaros et al. [10] conducted experimental measurements of reflective foils separated by layers of polyester wadding and polyester fibrous insulation. It consisted of the seven silver-coated foils with a measured emissivity of 0.2 for the exterior foils and 0.15 for the interior foils respectively. Awoyera et al. [11] enhanced the thermal resistance of EPS by integrating it with a Radiant Barrier Foil Board (RBFB) material, thus forming a composite insulation material. Results show that the EPS-RBFB composite demonstrated a 95% reduction in emissivity level and a 22% improvement in thermal conductivity.

Jia et al. [12] developed a material with ultra-low infrared emissivity (0.035 in the band of 8–14 μm) on the base of well-dispersed aluminum powder and resin. In turn, the authors of [13] presented a non-metallic material that has better reflective properties than traditional materials. Stevens et al. [14] introduced a concept of dynamic radiant barriers intended for residential attics, which can switch between reflecting and transmitting states as needed.

Cheng et al. [15] significantly expanded the scope of analysis of low-emission materials in the infrared by adding aspects related to their color and transparency in the visible range. Wang et al. [16] developed a retro-reflective coating (RR-coating) that reflects radiation along the incident direction. Research is also underway on materials that combine different functions. G. Zhang et al. [17] developed a material with a reflective surface and a porous, insulating structure with a low thermal conductivity coefficient.

Saber et al. [18] conducted numerical modeling and experimental investigations to assess the thermal performance of assemblies with reflective insulations. Two tests were conducted on sample stacks using the heat flow meter apparatus FOX 314. The measured, non-uniform, heat fluxes did not represent the actual heat passing through the whole sample stack. This observation should be taken into account when assessing and comparing the results of plate apparatus tests with real structures. In their next paper [19] the authors paid attention to not only the effect of air intrusion on air gaps and the resulting change of RI resistance but also possible dirt deposition and water vapor condensation. Similar issues were analyzed in [20].

In a real building with finite dimensions of air space, 2D heat flow should be considered. It was confirmed again that single air spaces with heat flow increase R-value changes significantly, with a change in the aspect ratio for the full range of the effective emittances [21].

Gerson et al. [22], in their mathematical model, not only considered combined two-dimensional heat but also air and moisture transport through unsaturated roofing systems. Fricker et al. [23] reviewed and discussed four methods of calculating R-values of the enclosed reflective air spaces, and one of them was a procedure contained in Annex D of ISO 6946 [24]. The achieved results generally agree to within 10%. Malz et al. [25] presented the results of computer simulations aiming at an evaluation of the influence of long-wave infrared reflecting (LWIR) wall paint on energy saving potentials and human thermal comfort. A similar problem was studied by Wille et al. [26]. They combined IR heaters with IR reflective walls and proved that in such conditions less radiant heat is absorbed by the surrounding walls and more is reflected to and absorbed by the occupants. This allows for lower air temperatures while maintaining constant thermal comfort.

A novel wall insulation system implying wooden fiberboards and intermediate air layers with highly reflective interfaces was introduced in [27]. The structure of the multilayer insulation consisted of six air layers with a thickness of only 1 cm. According to the authors, the multilayer insulation concept is able to compete with state-of-the-art insulations; therefore, an effective thermal conductivity of equal to or lower than 0.035 W/(mK) should be reached.

Radomski et al. [28] confirmed that application of the FOX 314 apparatus for determining the thermal conductivity of reflective insulations gives substantially reliable results, although other real factors, like moisture or solar radiation, cannot be considered. Pourghorban et al. [29] conducted full-scale testing of reflective insulation systems based on bubble foil with a guarded hot box apparatus in order to compare their results to the ISO 15099 [30] calculation method. The results prove that the resistance values for reflective air spaces derived from the ISO standard were much higher than the measured ones. The authors listed possible reasons of the observed discrepancy: a lack of uniformity and leveling resulting in convective air flows, negligence of thermal bridges and imperfect airtightness of air gaps.

In the study [31], a steady-state numerical simulation approach using ISO 15099 was utilized for performing calculations in enclosed reflective air spaces. The authors proved the importance of evaluating reflective insulation systems under actual operational conditions in different climates, instead of taking a constant R-value. The results of a newly developed multilayer reflective insulation system called mirror-panel testing were presented in [32]. The mirror-panel samples consisted of layers of aluminum foil (emissivity of 0.11) and paper coated with low-emissivity paint (0.52), separated by air spaces of approximately 5 mm. Changes in thermal resistance were investigated depending on temperature, in the range from 0 to 35 °C. According to the authors, this change is not due to changes in emissivity but to the strong dependence of heat transfer by radiation on temperature and air convection intensity.

Simpson et al. [33] authored a paper on the thermal performance of thermal paints and thermal coatings, which are often advertised as good insulators that also block radiative heat transfer. Due to aggressive advertising, easy paint application, and relatively low prices, many building users apply such materials. However, evidence from the achieved results, as well as scanning electron microscopy, does not support the claim that the coatings have low-emissivity surfaces. The lowest long-wave emissivity value of thermal paints that they tested was 0.72, and the highest was 0.89. So, in fact, they are all high-emitting materials.

Experimental, computational and simulation studies of reflective isolation were carried out by Escudero et al. [34]. What is very important in this context is that the authors used a unique method of emissivity testing in a plate apparatus. Results obtained by them of calculations according to the EN ISO 6946 standard [24] and laboratory tests were in good agreement. Barreira et al. [35] evaluated the emissivity of building materials using two common approaches: a specialized apparatus emissometer and the comparative method. Both measurement methods produced satisfactory results for conventional high-emissivity building materials, and significant discrepancies were observed for metallic surfaces.

Alev et al. [36] compared the insulating effects of 66 mm-thick mineral wool and a system consisting of a 10 mm-thick insulating mat with reflective coatings and 25 mm closed air gaps. The thermal resistance of both solutions was found to be similar, and the obtained result was totally inconsistent with manufacturer information, according to which reflective insulation thermal resistance is equivalent to 200 mm mineral wool.

According to the above review and the author’s own observations, the way reflective insulation works is not widely understood, not only by building users but even by people involved in the building industry. This, in turn, creates opportunities for misunderstandings, false interpretations of physical phenomena, and unreal declarations. These observations are based mostly on the false statements that appear in advertising materials or even popular science publications. In the event of obvious discrepancies between measured and declared insulating properties, some manufacturers claim that the physical phenomena occurring in these materials cannot be measured using conventional methods due to the mysterious “heat reflection.” Sometimes, instead of sound laboratory testing, results of comparative studies on models or in existing buildings are provided, which are difficult to analyze and impossible to verify or replicate.

Exactly the same opinions may be found in the papers authored by Alev et al. [36] and Hauser et al. [37]. They reported that some manufacturers of infrared reflective insulation products claimed that their relatively thin products feature the same high thermal performance as 20 cm mineral wool and maintained that their products cannot be described by conventional methods of building physics, and their thermal efficiency can only be determined by in situ tests. The research conducted by Hauser [37] in laboratory and in situ confirmed that general testing of reflective insulation can be successfully conducted in laboratory conditions. And what is equally important is that it is not possible to replace laboratory testing solely with in situ testing due to variable weather conditions and the numerous factors that influence test results in real environments. Cardona [38] proposed a cost-effective alternative of comparative analysis for industrial applications. The applied primarily qualitative method that utilizes an infrared lamp allows one to assess thermal emissivity and other thermal properties.

The above presented literature information and conducted research formed the basis of this paper. Various solutions and new reflective materials are being intensively developed and tested, but the key initial aspect is the actual emissivity of the material surface. The author’s primary goal is to provide the easiest yet reliable way to test the actual emissivity of materials available on the market. The hypothesis tested here concerns not only the basic qualitative approach but also the quantitative potential of the proposed method. The next hypothesis concerns the limits of the insulating capabilities of reflective materials compared to typical insulating materials. It is expected that the results will indicate achievable insulating effects and allow for the verification of false expectations or promises. For this purpose, a testing procedure is proposed which can be carried out in a typical building laboratory. It was used to verify the quality of several reflective insulation products available on the building market. Laboratory measurements and comparisons with the ISO standard procedure [24] allowed for the approval of the proposed procedure and the formulation of a number of conclusions regarding practical applications of reflective insulation. The obtained results made it possible to evaluate the actual thermal performance of reflective insulation composite systems relative to conventional insulation materials and to draw conclusions regarding the effectiveness and suitability of reflective insulation in different construction sectors.

## 2. Comparative Method of Long-Wave Emissivity Measurement

This section presents a detailed procedure for determining the long-wave infrared emissivity of building materials using the comparative method in accordance with EN ISO 6781-1:2023 [39]. The applied measurement principle, experimental setup, and environmental conditions required to ensure reliable results are described in detail.

Low infrared emissivity (*ε*) is a key characteristic of reflective insulation materials. Emissivity is defined as the ratio of thermal radiation emitted by a real surface to that emitted by an ideal blackbody at the same temperature. Most conventional building materials exhibit relatively high emissivity values, typically ranging from 0.50 to 0.95. In contrast, chemically clean and polished metallic surfaces may exhibit emissivity values that are several times lower than those of common construction materials. In building applications, aluminum is most frequently used to achieve low-emissivity surfaces, either in the form of thin foils or vacuum-deposited metallic coatings combined with polymer substrates and protective layers. However, recent studies have also explored the development of non-metallic materials with comparable reflective properties [13].

Accurate determination of emissivity generally requires specialized laboratory equipment, such as hemispherical blackbody-based infrared measurement systems. Commercial emissometers are available as both laboratory instruments and portable devices; however, access to such equipment is typically limited to specialized research facilities. Practical emissivity measurements can nevertheless be performed using simplified methods with acceptable accuracy. Annex B of EN ISO 6781-1:2023 [39] describes two field procedures for emissivity determination, one of which is the comparative method adopted in the present study.

The comparative method is based on thermal imaging and the use of a reference material with known emissivity. A certified reference material, typically in the form of a thin self-adhesive tape, is characterized by a manufacturer-specified emissivity value. A piece of the reference tape is attached to the surface of the test specimen, which is heated to a temperature significantly above ambient conditions. Owing to its negligible thermal resistance, the reference tape is assumed to have the same surface temperature as the underlying test material.

The surface temperature of the reference area is first measured using a thermal imaging camera with the emissivity of the reference material entered into the camera settings. Subsequently, the camera is directed towards the adjacent tested surface, and the emissivity setting is manually adjusted until the measured temperature of the test surface matches the previously determined temperature of the reference area. The emissivity value corresponding to this condition is taken as the emissivity of the investigated material.

**Figure 1:**
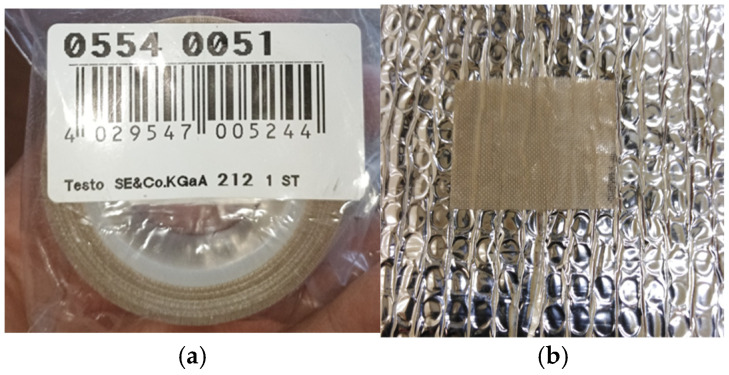
(**a**) Certified reference tape with a long-wave emissivity of 0.95; (**b**) tested sample with reference tape applied.

The described procedure was implemented at the Building Physics Laboratory of Cracow University of Technology. A certified Testo (Testo SE & Co. KGaA, Schwarzwald, Germany) self-adhesive reference tape with a long-wave emissivity of 0.95 was used throughout the study (Figure 1).

To minimize measurement errors, the test specimens were shielded from local sources of thermal radiation. The thermal imaging camera compensates for background radiation by accounting for ambient temperature. All measurements were conducted under identical laboratory conditions, with an indoor air temperature of 24 °C and relative humidity between 45% and 50%. During testing, all artificial lighting was switched off, the window was covered with an opaque blind, and the experimental setup was additionally screened from the external wall and window using a reflective barrier.

Under these conditions, the mean radiant temperature was assumed to be equal to the indoor air temperature. Prior to testing, all samples were cleaned with isopropyl alcohol to remove surface contaminants. No additional surface treatments were applied, ensuring that the tested materials remained representative of their intended practical applications.

For low-emissivity materials, maintaining a surface temperature significantly above ambient conditions is particularly important, as this reduces the influence of reflected environmental radiation on the measurement result. Consequently, the experimental setup was designed to provide stable thermal conditions while ensuring uniform temperature distribution between the reference tape and the test surface.

**Figure 2:**
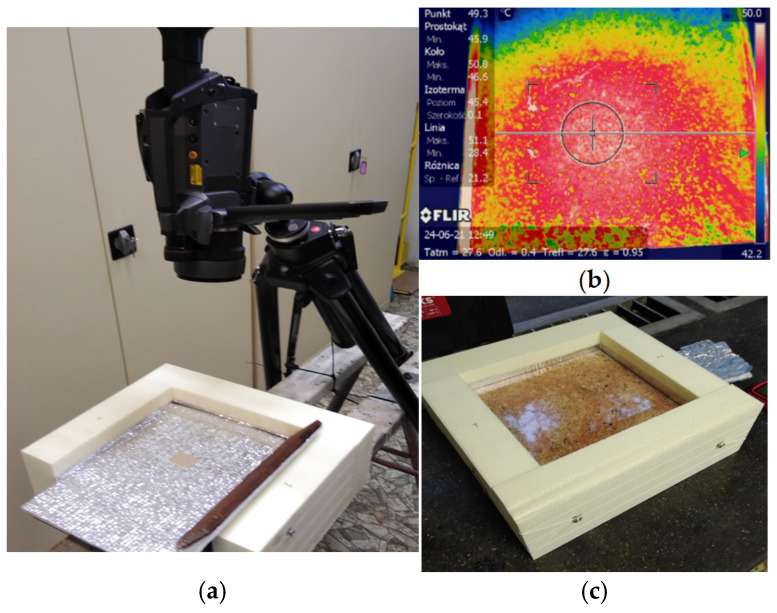
(**a**) Measurement station with a FLIR ThermoCAM P660 infrared thermal imaging camera; (**b**) thermal image of a granite slab surface; (**c**) insulated granite slab covered with glass.

Instead of using electrically heated metal plates, which typically exhibit cyclic temperature fluctuations, a massive granite slab measuring 24 × 24 cm and 3 cm in thickness was employed as the primary heat source (Figure 2a,b). Prior to testing, the slab was heated in a laboratory oven to approximately 65 °C. To reduce heat losses, five sides of the slab were insulated with thick layers of extruded polystyrene (XPS). The selected temperature was sufficiently low to prevent thermal degradation of the insulation.

The crystalline and heterogeneous structure of granite produces non-uniform thermal patterns that may affect surface temperature distribution. To minimize this effect, a 4 mm-thick glass plate was placed on top of the granite slab during testing (Figure 2c). The large thermal mass of the insulated assembly ensured slow and nearly monotonic cooling, thereby providing stable measurement conditions and facilitating accurate emissivity determination.

The accuracy of the emissivity measurements depends strongly on compliance with the required environmental conditions and on the performance of the thermal imaging system. Although the overall measurement uncertainty remains difficult to quantify, particularly for materials with very low emissivity, the method provides a practical means of distinguishing genuinely reflective materials from conventional high-emissivity building products. The primary objective of the procedure is therefore not to replace laboratory-grade emissivity measurements but rather to provide a reliable and accessible method for preliminary verification of material performance and manufacturer declarations.

A FLIR (Teledyne FLIR, Wilsonville, OR, USA) ThermoCAM P660 infrared camera was used for all measurements. The camera features a thermal sensitivity of 0.04 K, an image resolution of 640 × 480 pixels, and a measurement accuracy of ±2 °C or ±2% of the recorded value. The instrument operates within the long-wave infrared spectral range of 7.5–13 μm. During testing, the camera was positioned approximately 80° relative to the sample surface to minimize reflections originating from both the camera and the operator.

The uncertainty of emissivity measurements performed using an infrared camera depends on several factors, including the temperature accuracy of the camera (sensor calibration), the surface temperature, the reflected apparent temperature, and the optical properties of the investigated material. For high-quality infrared cameras, the specified temperature measurement accuracy is typically ±2 °C or ±2% of the measured value. In the comparative method employed in this study, this uncertainty constitutes the primary source of measurement error, as it affects both the reference temperature measurement and the determination of the infrared radiation emitted by the test specimen. Therefore, it is important to use a camera with the lowest possible measurement uncertainty for testing.

For high-emissivity surfaces, a temperature uncertainty of ±2 °C typically corresponds to an emissivity uncertainty of approximately 0.01–0.02, resulting in a relatively small relative error. In contrast, for low-emissivity materials (*ε* < 0.2), the emissivity uncertainty may exceed 0.1 because of the increased contribution of reflected ambient radiation. The uncertainty cannot be quantified more precisely, as it also depends on the temperatures of both the measured surface and its surroundings. In general, increasing the temperature difference between the object and the environment reduces the influence of reflected radiation, thereby improving the accuracy of emissivity determination.

These considerations indicate that the thermal characterization of reflective insulation systems based on indirect emissivity measurements is inherently associated with significant uncertainty and therefore requires independent validation. In the present study, the obtained emissivity values were verified using a two-stage procedure. First, the thermal resistance of the reflective insulation system was determined experimentally using a guarded hot plate apparatus. Second, the measured thermal resistance was compared with the value predicted by the standardized calculation method. This procedure enables the thermal performance of the complete reflective insulation system to be evaluated from the known thermophysical properties of its constituent layers, including emissivity, thermal conductivity, and thermal resistance.

**Figure 3:**
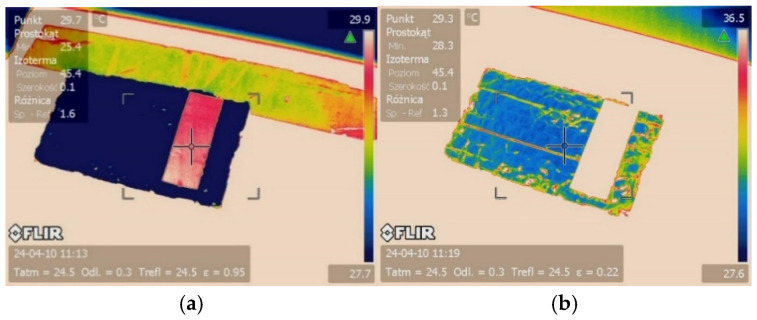
Method of comparative measurement of the long-wave emissivity of the reflective insulation surface: (**a**) Temperature measurement of the reference tape with emissivity set to 0.95; (**b**) thermal image of the foil with an assumed emissivity of 0.22.

Figure 3 illustrates a representative measurement procedure. The high-emissivity reference tape appears as a small rectangular area attached to the surface of a reflective foil specimen. When the camera emissivity setting is maintained at 0.95, the low-emissivity foil appears significantly cooler than the reference area and falls outside the lower limit of the selected temperature scale. By reducing the assumed emissivity value to approximately 0.22, the measured temperature of the foil becomes consistent with that of the reference area, indicating that the actual emissivity of the foil is close to this value.

Despite the relatively high temperature of the heating plate, the surface temperature of multilayer reflective insulation materials typically remains within the range of 30–40 °C, which corresponds to temperatures commonly encountered in building applications. The authors of [40,41] proved that temperatures within this range do not significantly affect the reflective performance of such insulation materials.

## 3. Emissivity Measurement Results

Experimental verification of actual emissivity, rather than reliance solely on manufacturer declarations, is essential for the proper assessment of radiant barrier performance. However, the literature review conducted by Barreira et al. [35] shows that there is no consensus on the procedure for determining the emissivity of construction materials. Consequently, the ability to verify emissivity values using equipment commonly available in building physics laboratories is of considerable practical importance. It should be emphasized that strong reflectance in the visible spectrum, as perceived by the human eye, does not necessarily correspond to high reflectance in the long-wave infrared range. For example, covering an aluminum surface with a thin layer of transparent polymer may have little effect on visible-light reflectance while substantially reducing its ability to reflect thermal radiation.

The comparative measurement procedure described in the previous section was applied to determine the emissivity of five materials commercially available on the construction market and marketed by their distributors as reflective insulation products. The measured long-wave emissivity values are summarized in Table 1.

For material RI-1, the manufacturer specified that both outer surfaces consisted of the same reflective layer; therefore, only a single emissivity value was determined. For the remaining products, no detailed information regarding the surface layers was available, and consequently both sides of each material were tested separately. In addition, emissivity measurements were performed on both sides of conventional household aluminum foil. The material designations presented in Table 1 are intended solely for identification purposes and do not constitute product trademarks.

The results indicate that only two of the tested building products exhibit genuine infrared low-emissivity properties and are capable of effectively reflecting long-wave infrared radiation. The remaining materials, despite their metallic or glossy appearance, exhibit emissivity values characteristic of conventional building materials and therefore provide little or no reflective performance in the thermal radiation range.

**Figure 4:**
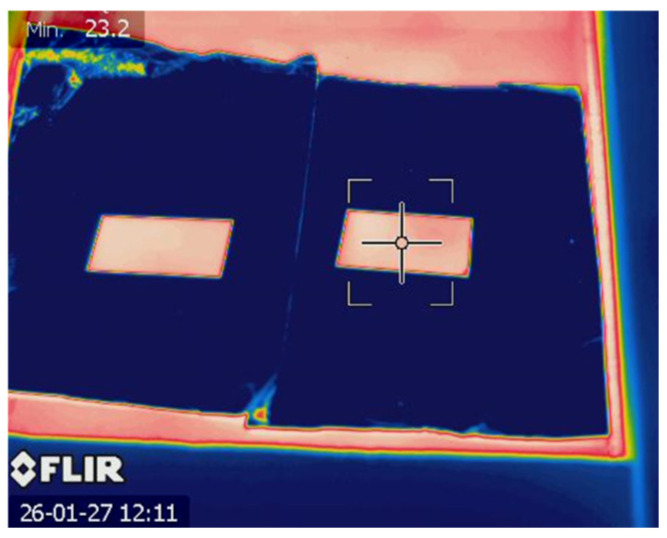
Thermal image of household foil samples; on the left side is the mat surface, on the right side is the glossy surface, and the same emissivity was set in the camera as for the reference tape strips (*ε* = 0.95).

Conventional household aluminum foil demonstrated excellent emissivity values. To ensure uniform thermal contact with the heated substrate, thin foil specimens were attached using thermal paste, thereby eliminating air gaps between the sample and the substrate. Emissivity measurements performed on both sides of the foil yielded nearly identical results. Thus, the visible differences between the glossy and matte surfaces did not result in measurable differences in long-wave infrared emissivity (Figure 4).

A significant practical challenge associated with the application of reflective insulation is the gradual deterioration of surface properties resulting from aging and the accumulation of dust and other contaminants during construction and long-term service life [2]. Lee et al. [1] identified four primary factors contributing to an increase in emissivity: dust deposition and surface contamination, moisture condensation, corrosion, and oxidation. This issue is particularly important for reflective insulation systems intended to remain exposed within building interiors or attic spaces, such as those used in lightweight industrial roofing systems, attic insulation, and radiator reflective screens.

Although complete prevention of surface degradation is difficult, every effort should be made to protect reflective surfaces from dust accumulation and moisture exposure. Appropriate design measures, maintenance procedures, and periodic cleaning may help preserve the low-emissivity characteristics of reflective insulation throughout its service life [1].

## 4. The Thermal Resistance of the Compound Reflective Insulation

### 4.1. The Method of Measurement in a Plate Apparatus

The most commonly used reflective building materials take the form of a complex system of foils and flexible porous or bubble materials with a low thermal conductivity coefficient (λ). Reflective layers may be used as spacers between insulating layers, as well as on the edges of the entire assembly. Regardless of the arrangement and properties of the individual layers, the main commercial information for the user is the expected thermal resistance of the entire composite product. However, technical information provided by manufacturers of composite reflective insulation comes in various forms. This may include the thermal resistance of the standard insulation alone, the resistance of the insulation with air gaps or the equivalent thermal conductivity of the entire system. Primarily for marketing purposes, manufacturers sometimes provide the equivalent thickness of traditional thermal insulation.

The basic experimental method of RI thermal testing, according to standards [42], involves measurements conducted in a climatic chamber using the Hot Box method, under conditions consistent with the intended use of the insulation material. However, these measurements are time-consuming and expensive due to the size of the samples and the entire device. The second approved method concerns the measurement of the thermal resistance of a reflective system using a plate apparatus, commonly used for thermal conductivity testing. For materials with reflective external facings, air cavities must be created around the edges to account for the effect of reduced emission and absorption of radiation.

**Figure 5:**
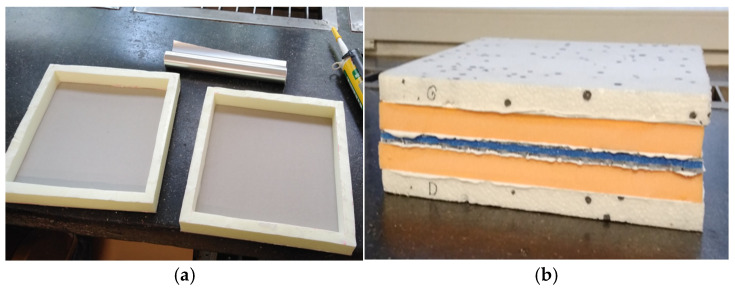
Preparation of reflective insulation for testing in the FOX 314 apparatus: (**a**) Two XPS frames creating air cavities; (**b**) the whole tested assembly: in the middle tested sample between the XPS frames, white insulation boards were on the top and bottom.

**Figure 6:**
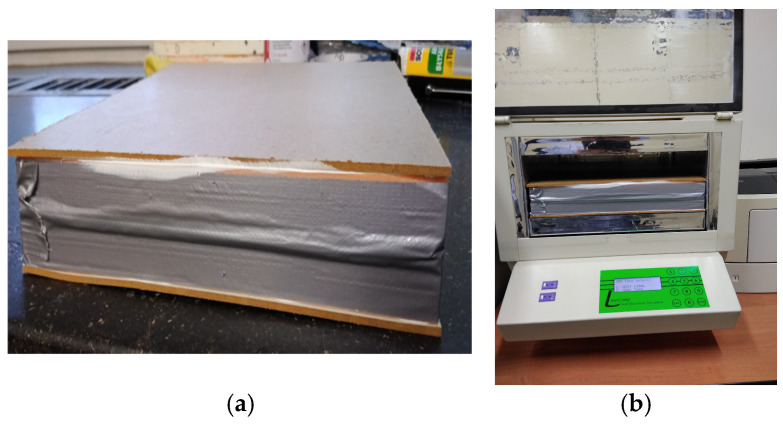
Reflective insulation testing: (**a**) Reflective insulation between two XPS frames, with the entire package wrapped tightly with tape; (**b**) sample in the FOX 314 apparatus during thickness measurement.

Figure 5 and Figure 6 show how reflective insulation samples were prepared for measurement in the FOX 314 plate apparatus, produced by TA Instruments, New Castle, DE, USA. This apparatus tests samples measuring 30 × 30 cm and a maximum thickness of 10 cm. The measurement field in the central part of the sample is 10 × 10 cm. The uncertainty of the thermal conductivity measurement is 1% in the range of 0.01–0.2 W/(mK).

The tested samples were mounted between two XPS frames using a fast-curing acrylic adhesive. The air cavities formed within the frames were enclosed on the top and bottom surfaces with insulating panels, and the complete assembly was sealed along its perimeter using impermeable adhesive tape (Figure 5). The sample sealing serves to separate convection phenomena within both air spaces. The edge insulating layers cover the metal plates of the apparatus, with unknown emission properties. Furthermore, the factory-applied coating on the plates is subject to abrasion during intensive use, creating measurement uncertainty that is difficult to determine. Top and bottom insulating boards eliminate this uncertainty. The thermal resistance of the shielding materials was measured separately and considered in the calculation of the reflective insulation resistance in each case.

### 4.2. Reflective Compound Insulation RI-1

**Figure 7:**
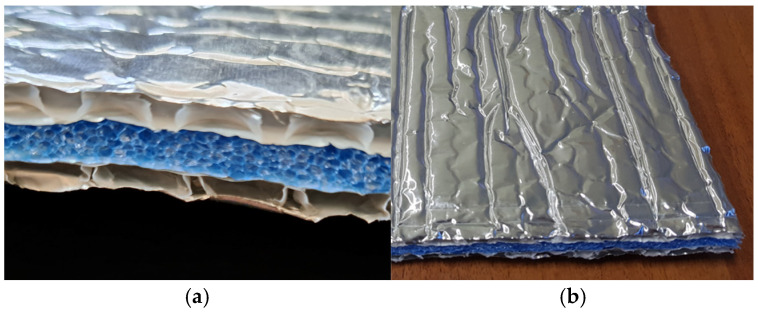
(**a**) Cross-section of reflective insulation; (**b**) surface view of RI-1 material.

The CUT laboratory measured the thermal resistance of the composite reflective insulation RI-1 using the plate apparatus FOX 314. According to EN ISO 22097 [42], this material can be classified as Type 1 reflective insulation. The total thickness of this material, excluding external air gaps, is 10 mm. According to the manufacturer, it consists of a total of seven layers: two layers of pure, polished aluminum that are 30 microns thick, protected against oxidation, separated by two layers of air bubbles in a honeycomb structure, and encased in a polyethylene film and a core of self-extinguishing blue polyethylene foam, Figure 7. Additionally, it contains two outer reflective layers of pure aluminum. The manufacturer claims that the emissivity of the foil is lower than 0.05, and with a thickness of 10 mm and the two separated air gaps, this product achieves a calculated thermal resistance of 5.70 m^2^K/W and is “up to 13% more efficient than 20 cm of mineral wool”.

The emissivity of the outer surfaces of this multilayer material was previously tested by the author (Table 1) and averages 0.1. Therefore, despite the differences in the information provided, both surfaces of this material have true reflective properties.

The RI-1 insulation test began with measuring the thermal resistance of the insulation core itself, without surrounding air gaps. Measurements were performed in various configurations: the sample itself and a sample shielded from the apparatus plates by materials with known thermal resistance. The average thermal resistance value from the three measurements is 0.296 m^2^K/W, with a maximum value spread of 13%. The equivalent thermal conductivity of the combination of foam layers and aluminum foils is 0.034 W/mK. Therefore, the possibility of obtaining a total thermal resistance equivalent to 20 cm of mineral wool can already be ruled out at this point. As a result of further testing, the thermal resistance of the insulation with two air gaps, each nominally 20 mm thick, with heat flow from the bottom upwards was 0.847 m^2^K/W. With reverse heat flow, the obtained thermal resistance was 1.084 m^2^K/W. According to the results, the thermal resistance of the reflective insulation system and two air gaps is more than five times lower than the manufacturer’s specifications. The equivalent thermal conductivity of the entire 5 cm-thick system is 0.059 W/mK, i.e., is higher than the standard thermal insulation conductivity.

The material described above received a technical certificate called the National Technical Assessment, issued by the Building Research Institute [43]. According to this assessment, the thermal resistance of the entire RI-1 insulation system and two air gaps, at least 2 cm thick, is 0.878 m^2^K/W, with heat flowing vertically upwards. The difference in thermal resistance values obtained in the tests conducted by the author and the national research institute for upward heat flow is only 3.7%. This official document therefore confirms the multiple discrepancies between the manufacturer’s declared value and the actual value, and at the same time, it also confirms the correctness of the measurements described above.

RI-1 reflective insulation was also tested with air gaps thickened to 29 and 35 mm, with heat flow from the bottom to the top. For 29 mm-thick gaps, an increase to 0.978 m^2^K/W in thermal resistance of the entire system was observed, while with a gap thickness of 35 mm, the thermal resistance of the entire system decreased to 0.798 m^2^K/W.

### 4.3. Reflective Compound Insulation RI-2

**Figure 8:**
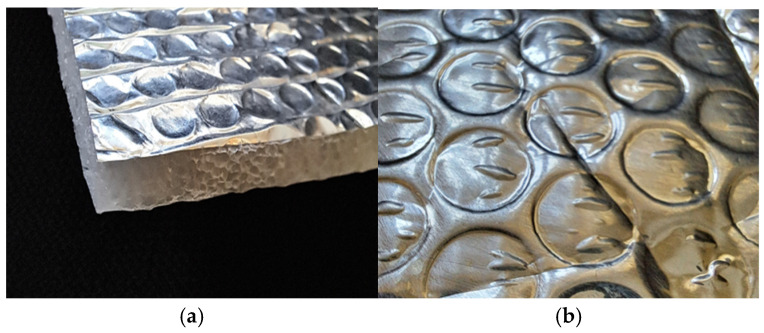
(**a**) Cross-section of reflective insulation; (**b**) surface view of RI-2 material.

The second material tested in the CUT laboratory was polyethylene foam that was approximately 6 mm thick and coated on both sides with reflective foil. Regardless of the shape of the embossing on the foil surface (Figure 8), this material does not have an internal, regular, bubble-like structure. As shown in previous studies (Section 3, Table 1), the long-wave emissivity of both sides of this material is quite high (0.68); so, in this case, it is difficult to expect a significant impact of both surface layers on the thermal resistance of the entire system.

The measured thermal resistance of the insulation core itself was 0.161 m^2^K/W. This material was then placed between two closed air gaps, each 20 mm thick, and the heating plates were covered with m^2^K thin cardboard with an emissivity of 0.85. The thermal resistance of the entire system increased to 0.552 m^2^K/W with bottom–up heat flow and to 0.576 m^2^K/W with reverse heat flow. For 29 mm-thick gaps and bottom–up heat flow, the measured thermal resistance was slightly lower, at 0.541 m^2^K/W.

Based on the obtained measurements, the partial thermal resistances of the air gaps themselves were calculated. The total resistance of both gaps, equal to the difference between the total resistance and the resistance of the foam insulation core, is 0.391 m^2^K/W. Theoretical calculations of the thermal resistance of a horizontal gap with bottom–up heat flow were also performed, according to the frequently used and readily available procedure in the EN ISO 6946 standard [24]. The following assumptions were made: the temperature difference across the gap is greater than 5 K, the average temperatures in air gaps are 4 °C and 16 °C respectively, and the measured emissivity coefficients are 0.85 and 0.68. Under these assumptions, the calculated thermal resistance of the colder horizontal gap is 0.196 m^2^K/W, and that of the warmer one is 0.181 m^2^K/W. The difference between the calculated and the measured values of gap thermal resistance is 3.7%. However, if the surface of this insulation were covered with highly reflective materials, for example, with an *ε* coefficient of 0.05, the resistance of the colder gap would be 0.414 m^2^K/W, and that of the warmer gap would be 0.408 m^2^K/W, which is more than twice as high. Reflective material would allow for a 78% higher resistance of the entire insulation system at the same thickness. Therefore, a designer or user of this material who is unable to verify the actual properties of the material and accepts advertising information in good faith will in reality obtain insulation performance values that are a few times lower than desired. What is important is that the obtained very closely measured and calculated values confirm the proposed assessment procedure of emissivity and thermal resistance testing.

### 4.4. Reflective Compound Insulation RI-3

The third material tested was a thin film that was approximately 3.2 mm thick. Due to the high compressibility of this product, more precise thickness determination is difficult. It is a flexible material containing a single layer of small air bubbles, coated on both sides with foil. The previously tested emission properties of both foil surfaces are 0.59 and 0.66, respectively.

**Figure 9:**
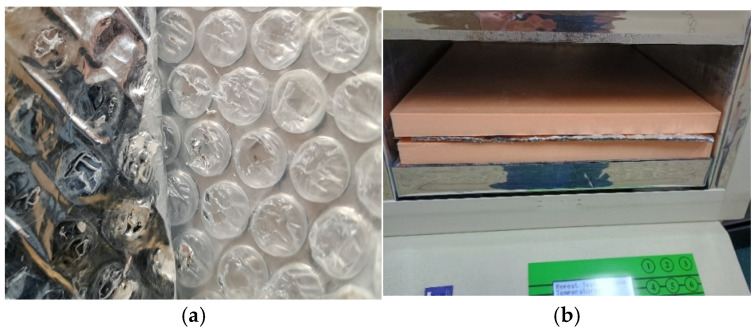
RI-3 foil: (**a**) Bubble core layer; (**b**) RI-3 foil (no air gaps) tested in the FOX 314 apparatus.

The thermal resistance of the foil only, measured in the FOX apparatus (Figure 9), is 0.108 m^2^K/W. Testing this material with 20 mm-thick air gaps yielded a total resistance of 0.503 m^2^K/W, with the upward heat flow. The total thermal resistance of the two horizontal air gaps is 0.395 m^2^K/W. So, there is no significant reduction in radiative heat transfer in the air gaps.

The thermal resistance of both gaps, calculated according to EN ISO 6946 [24], assuming that the emissivity of both surfaces is equal to the measured values given above, is 0.387 m^2^K/W. The difference between the measured and calculated values in this case is only 2.1%, thus confirming the overall value of the measurements and the lack of significant reflective effects of this material.

If the emission properties of both surfaces were 0.05, the resistance of the entire system would be equal to 0.928 m^2^K/W. However, such resistance cannot be achieved using the RI-3 material in its current form.

### 4.5. Reflective Compound Insulation RI-4

**Figure 10:**
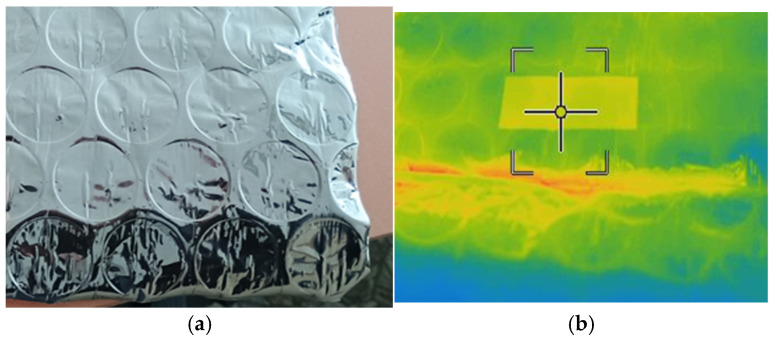
RI-4 material: (**a**) Top shiny layer of foil; (**b**) thermal image of foil and reference tape, with emissivity set in the camera to 0.95.

The fourth type of insulation is a foil P with large (3 cm) bubbles, which is 6.6 mm thick and metallized on one side with aluminum. Although the foil has an intensely shiny surface (Figure 10), its average emissivity coefficient, given in Table 1, is 0.63. The thermal image confirms that the emissive properties of the metallized surface of this material are close to those of the reference tape.

The bubble structure of this material is clearly visible in the thermal imaging image. The measured thermal resistance of the insulation alone, without air gaps, is 0.198 m^2^K/W. For further testing in the plate apparatus, the foil was placed with the reflective side facing the warm heating plate, i.e., at an average air temperature of approximately 16.5 °C. The thermal resistance of the entire system (RI-4 insulation and two 20 mm air gaps each) with heat flow from the bottom to the top is 0.532 m^2^K/W. Therefore, the measured total thermal resistance of the two air gaps with surfaces of different emissivity values is 0.334 m^2^K/W.

The temperature difference across the gap thicknesses exceeds 5 K; so, the formulas in Table D.2 of the [24] standard were used in the computational analysis. The thermal resistance of a 2 cm horizontal air gap, at an average temperature of +16.5 °C and surface emissivity of 0.90 and 0.63, is 0.189 m^2^K/W, according to the standard. The calculated resistance of the second air gap, with an average temperature of approximately +3 °C and emissivity of both surfaces of 0.90, is 0.178 m^2^K/W. The total calculated resistance of both gaps is 0.351 m^2^K/W, which is 5% higher than the measured value.

It should be stated again that the tested building material does not have significant low-emission properties in the infrared range and cannot be considered a reflective insulation material.

### 4.6. Reflective Compound Insulation RI-5

**Figure 11:**
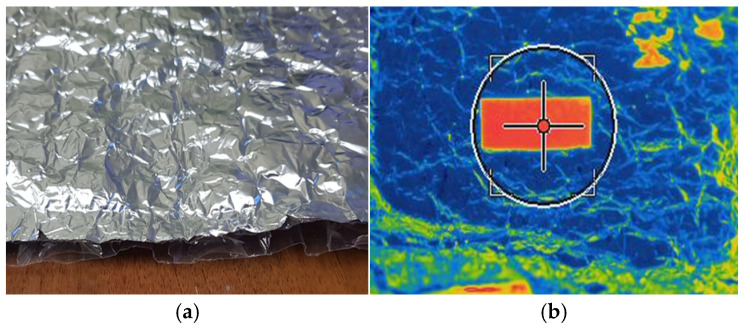
Insulation RI-5: (**a**) View of the material structure; (**b**) thermal image of the foil and reference tape, with the camera set to an emissivity of 0.95.

The fifth type of insulation analyzed in this article is foil M with large bubbles, which is approximately 7.6 mm thick and metallized on one side with aluminum (Figure 11). Compared to the RI-4 material, the surface of the RI-5 foil is not completely smooth; the metallic coating underwent permanent deformation. A visual comparison of the reflective surfaces of the RI-4 and RI-5 materials suggests significant similarity. However, the fundamental difference between these materials concerns their reflective properties in the infrared range. The average emissivity coefficient of the shiny surface of the RI-5 foil, as shown in Table 1, is 0.05 and corresponds to the properties of a pure aluminum layer. The thermal image confirms the contrasting emission properties of this material compared to the reference tape.

The average thermal resistance of the foil alone, without air gaps, measured in the plate apparatus is 0.228 m^2^K/W. Due to the asymmetric structure of this material and the low emissivity of only one surface, the resistance measurement was repeated by reversing the material in the apparatus. The obtained difference in results is 0.5%, which is less than the manufacturer’s declared measurement uncertainty.

As before, for further testing in the plate apparatus, the foil was placed with the reflective side facing the warm heating plate. The thermal resistance of the entire system (insulation and two 20 mm air gaps) and for heat flow from the bottom to the top is 0.771 m^2^K/W, and the total thermal resistance of the two air gaps is 0.544 m^2^K/W.

The formulas in Table D.2 of the [24] standard were also used in the computational analysis. Due to the higher air gap resistance next to the aluminum layer, the temperature distribution in the tested system changed. The thermal resistance of the horizontal air gap, at an average temperature of +15.0 °C and surface emissivity of 0.90 and 0.05, is 0.367 m^2^K/W, according to the standard. The calculated resistance of the second gap, with an average temperature of approximately +2 °C and emissivity of both surfaces of 0.90, is 0.172 m^2^K/W. The total calculated resistance of both air gaps is 0.539 m^2^K/W, and the difference in relation to the measured value is less than 1%.

### 4.7. Radiant Barrier: Aluminum Foil

Additionally, thermal insulation tests were performed using a FOX 314 apparatus for a system of household aluminum foil and two air cavities. The apparatus plates were covered with cardboard layers during the test, and their thermal resistance was considered in the calculations. The measured total thermal resistance of both cavities with heat flow from the bottom to the top was 0.737 m^2^K/W. According to calculations consistent with the standard procedure, the resistance of the cavities with an emissivity of 0.05 and 0.06 respectively (Table 1) and with a temperature difference above 5 K is 0.751 m^2^K/W. The difference between the two values is 1.8% of the lower one. The thermal resistance of this system, measured with the heat flow direction reversed, is 1.694 m^2^K/W, while according to the standard calculations, its value is 1.376 m^2^K/W. The difference between the measured and calculated values is significant in this case. As stated in Section 4.1, the error in measuring such low-emissivity values of an aluminum surface using a thermal imaging camera is unknown and may have a significant impact on the calculated values. However, the good agreement of the results for upward heat flow does not indicate that the emissivity measurement precision itself is the cause of this discrepancy. The obtained difference may be due to the heterogeneity of heat fluxes across the entire measurement field, as indicated in the article [18]. Finally, this observation does not change the general significance of the obtained results, which confirm the very favorable reflective properties of aluminum foil and the general usefulness of such systems in building structures.

## 5. Tests with Scanning Microscope

The collected results and all the questions gathered during the above-described analysis of reflective insulation led the author to additional examinations of the selected materials using a reflective scanning microscope.

Surface microstructure tests were performed using a Bruker EVO Zeiss MA10 scanning electron microscope (Carl Zeiss Industrielle Messtechnik GmbH, Oberkochen, Germany) with EDS and BSDs. The tests were performed under variable vacuum (at a pressure of approximately 100 Pa) on unsprayed samples, with a working distance of approximately 10 mm. Additionally, the surface chemical composition was analyzed using the EDS method (semi-quantitative and qualitative).

**Figure 12:**
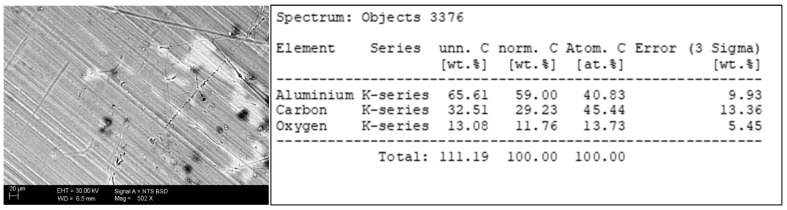
Material RI-1: Magnified image (500×) of the surface and print screen of the original report on the surface chemical composition.

The original report generated by the microscope software Bruker ESPRIT Suite 2 (Figure 12) provides a breakdown of the elemental composition of the tested sample with the following column designation:

- Element/series: the identified element and the specific X-ray shell.
- unn. C [wt.%]: unnormalized weight concentration.
- norm. C [wt. %]: normalized weight concentration; the percentage of each element by total mass.
- Atom. C % [at %]: atomic concentration; the percentage of each element by the total number of atoms.
- Error (3 Sigma) [wt.%]: statistical measurement error at the 99.7% confidence level.

The most important result of the examination was the percentage share of chemical elements identified by the detectors. The information recorded by this device concerns the limited surface layer, the thickness of which does not exceed 5 microns [44].

Three of the previously analyzed materials were examined using a scanning microscope: RI-1, RI-2, and standard household aluminum foil. It was expected that the significant differences in long-wavelength emissivity observed between the RI-1 and RI-2 materials would be confirmed by microscopic and scanning analysis of the material surfaces. The results of the household aluminum foil examination were intended as a reference level for comparison with the two building products.

Figure 12 shows a microscopic view of the RI-1 material surface at a 500× magnification and its approximate chemical composition, generated by electron spectral analysis. A fairly regular linear structure of the material’s surface, locally interrupted by small inclusions with a different chemical composition than the dominant surface, can be observed here. The most important element of the foil surface is aluminum, although it is not a completely homogenous metallic layer. The previously observed infrared reflective properties of this material are associated and explained by the significant (59%) aluminum content within the surface layer.

**Figure 13:**
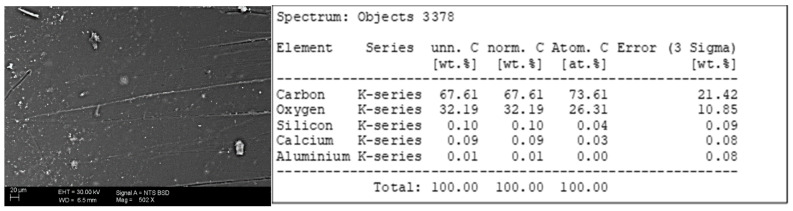
Material RI-2: Magnified image (500×) of the surface and print screen of the original report the surface chemical composition.

Figure 13 shows an image of the RI-2 material surface at the same magnification as before, along with a copy of the original report on the chemical composition of the surface layer. The dominant element is carbon, likely being a part of the structure of the polymers covering this material; virtually no aluminum was detected in the thin surface layer.

**Figure 14:**
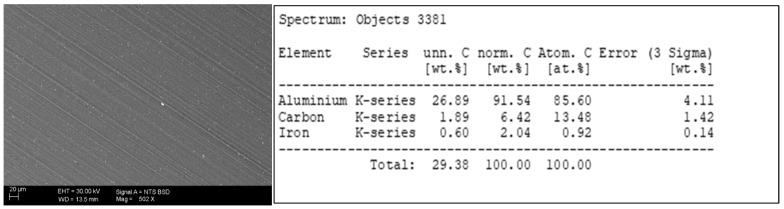
Aluminum household foil. Left: Magnified image (500×) of the surface. Right: Print screen of the original report on the surface chemical composition.

For comparison, Figure 14 shows a microscopic image of the shiny side of the household foil and an analysis of its chemical composition performed by a testing device. The barely noticeable presence of chemical elements other than aluminum may be due to factors such as minor industrial additives used in the foil production process, substances pressed in during foil rolling, surface chemical reactions of aluminum, or surface contamination.

The results of a chemical composition analysis of the tested materials using a Bruker EVO Zeiss MA10 scanning microscope are fully consistent with the previous measurements. Proportionally, the bigger aluminum content in the surface layer of the tested materials decreases its ability to absorb long-wave infrared radiation, while its reflective properties in this range increase. This explains the high reflective properties of the RI-1 material and the very poor reflective properties of the RI-2 material.

The RIMA guide [45] distinguishes two main types of commercial reflective products: Aluminum Foil Laminates and Aluminized Plastic Films—a thin layer of aluminum particles deposited on the film through a vacuum process. It can be assumed that in the case of the latter type of materials, even tiny details or changes in the technological process have a significant impact on the surface properties of the final product. According to the results of an electron analysis, the number of aluminum particles in such a product is too small, or they are covered with another material. The presence of aluminum particles coated even with a very thin (5-micrometer) layer of synthetic material does not translate into the expected infrared emission properties of the material’s outer surface, despite its highly reflective properties in the visible radiation spectrum. This observation is of paramount importance because it eliminates a significant portion of materials declared as reflective from the market.

## 6. Discussion

The comparative method employed in this study is not a basic physical approach for emissivity measurements, particularly in the case of very low-emissivity values. Nevertheless, owing to the availability of high-quality equipment and the simplicity of the procedure, the method was investigated as a practical tool for the characterization of reflective materials. The primary objective was to obtain a preliminary estimate of the emissivity range rather than an exact value accompanied by a rigorously quantified measurement uncertainty. However, as demonstrated by the tests conducted using the plate apparatus and by parallel calculations performed in accordance with the standard procedure, the adopted method provided results that were more accurate and consistent than initially anticipated.

Thermal testing of reflective insulation systems using devices equipped with heat flow meters (HFMs) or heat flow transducers (HFTs) is permitted by the relevant standard [41] and is widely applied because of the relatively low cost and broad availability of the equipment, the low testing cost, and the short measurement time [3,9,18,28,29,34]. Nevertheless, this approach has several limitations, including the restricted dimensions of the test specimens and the limited thickness of the systems that can be evaluated. Previous studies have also highlighted the challenge of non-uniform heat flux caused by convective air movement within air cavities [18,31]. This issue affects not only HFM- and HFT-based measurements but also hot box testing performed in climatic chambers. Therefore, these limitations should be carefully considered when interpreting results and designing testing procedures for reflective insulation systems.

The ISO standard procedure for calculating the thermal resistance of air cavities [24] is widely used in both research and engineering design. Based on the assumption of one-dimensional heat flow, the method has been shown to provide reliable results when compared with laboratory measurements performed using heat flow meter (HFM) apparatuses, as demonstrated both by the results presented in Section 4 and by previous studies, e.g., [23]. For the bottom-to-top heat flow configuration investigated in this study, the difference between the measured and calculated thermal resistance values ranged from 1% to 5%. In the author’s opinion, this close agreement between experimental and calculated results confirms the validity of the proposed methodology for evaluating reflective insulation systems. As defined in the standard [39], the measurement uncertainty of thermography in determining in situ thermal resistance, transmittance, and anomalies is very large, approximately +/− 25%. Although this value applies to the resistance of the entire component, not just the emissivity measurement, it provides some insight into the measurement challenges associated with thermal imaging. In this context, the obtained result can be regarded as encouraging.

A separate issue, which lies beyond the scope of the present study, concerns the limited applicability of laboratory test results to real building assemblies. In practice, air cavities have finite dimensions, complex geometries, and are bounded by structural elements characterized by relatively high emissivity and thermal conductivity. Consequently, an accurate assessment of heat transfer often requires two-dimensional thermal analysis [22]. Moreover, air cavities in actual building envelopes are rarely perfectly sealed, allowing for the ingress of water vapor and dust, which may significantly affect their thermal performance over time. Detailed investigations of these phenomena can be found in, among others, [19,20,23,29]. The authors suggest that it is not possible to assign a constant thermal resistance value without considering the local climatic conditions under which the material is used.

The ability of a material surface to emit and absorb long-wave infrared radiation is a fundamental parameter of reflective insulation. There is no simple relationship between surface properties in the visible and infrared ranges, which can be a source of errors or deliberate manipulation. Even articles on RI modeling or testing sometimes provide emissivity information based on manufacturer declarations without verification. This is not particularly important for experiments that finally verify the assumptions, but simulations based on false data are pointless. Saber et al. [18] cited the results of a study in which the emissivity of the foil bonded to the insulation was not measured. However, the authors stated that for “shiny metal surfaces,” the emissivity is approximately 0.05–0.1. Michels et al. [46] tested different reflective systems enabling the protection of a building against intensive heat gains through its roof. One of the solutions adopted 2 mm polyethylene sheeting with one surface advertised as reflective coating, but its long-wave emissivity was in fact higher than 0.5. These examples prove that basic information about infrared emission properties may be not very accurate, unreliable or even completely false. Barreira et al. [35] also highlighted the importance of measuring emissivity, since material characteristics and measurement conditions and procedures may influence results.

Confronted with commercial information from material manufacturers, the results of the tests presented in this article are surprising. For reflective insulation materials, the *ε* coefficient of the material’s surface is typically expected to be not higher than 0.2 [10,25,47]. However, the results summarized in Table 1 for three of the five tested materials deviate significantly from these expectations. So, in practice, with low thermal resistance of the core insulation layer itself and a lack of significant reflective properties, the actual thermal resistance of the entire system is very low.

The RI-1 reflective material, with good reflective properties, was tested also by Alev et al. [36] in natural conditions, in the cool climate of Estonia. The thermal resistance of the insulating core was equal to 0.279 m^2^K/W, which is 5.7% different from the value given in Section 4.2. The effect of the insulating properties of the entire system was shown by the authors [36] in the form of an equivalent thermal conductivity coefficient of 0.04 W/mK, with an air gap thickness of 2.5 cm. In Section 4.2, the laboratory measurement result was given as 0.059 W/mK, with gaps being 2 cm thick each. Both values correspond to a few centimeters of standard insulating material, while the manufacturer suggests that the thermal conductivity of the advertised material is several times lower, and its resistance is comparable to as much as 20 cm of typical insulating material.

Table 2 summarizes the equivalent thermal conductivity values of RI systems tested under the same conditions. The importance of the low-emissivity features of the surfaces of the materials RI-1, RI-5, and standard aluminum foil is clearly visible.

However, even low *ε* values do not allow us to expect values comparable to those of standard thermal insulation materials of the same thickness, even with air gaps of 2 cm or more. A similar relationship between RI and typical insulation materials is described in [4,29]. Other authors [7,27,32] suggest that only a combination of thin gaps and reflective surfaces allows for competitive insulating properties, while also reducing the cost and amount of embodied energy. However, such results are possible only with highly reflective aluminum layers (*ε* = 0.04).

A comparison of the thermal performance of reflective insulation (RI) and conventional insulation materials is commonly regarded as the primary criterion for assessing the suitability of RI in residential and similar buildings, where the available thickness of walls and ceilings is often limited. In roof spaces and attics, however, this constraint is generally less significant, and numerous studies have highlighted the high effectiveness of RI in reducing solar heat gains [5,6,9,22]. Consequently, a considerable body of literature has focused on the application of reflective insulation in roof retrofits of existing buildings located in warm and hot climates. Furthermore, Lee et al. [1] reported that RI can be a cost-effective solution when used in conjunction with lower levels of conventional thermal insulation in existing buildings.

Based on the results of the present study, the author suggests that the most promising applications of reflective insulation are lightweight structures with relatively low thermal-performance requirements that are currently uninsulated or poorly insulated, such as temporary warehouses, storage facilities, and the roofs of sports or entertainment arenas. An important advantage of RI, which receives comparatively little attention in the literature, is its very low weight. In structures with a limited load-bearing capacity, such as lightweight steel-framed buildings, the additional dead load imposed by conventional insulation systems may be a critical design constraint. Under such conditions, multilayer reflective insulation systems or radiant barriers may represent the only practical means of improving thermal resistance while fully satisfying structural limitations.

A detailed topic, only partially touched upon in this article, concerns the most favorable air gap thicknesses. The aforementioned thin (10 or even 5 mm) air gaps effectively limit the thickness of the entire insulation system, but they usually do not guarantee optimal resistance. Suggestions for the most favorable air gap thicknesses in terms of thermal resistance depend on the direction of heat flow relative to gravitational forces. The author performed only random tests of the effect of gap thickness on resistance, and the thinnest gap tested was 20 mm. In the case of the reflective material RI-1, a thicker gap (29 mm) caused only a 3.2% increase in the resistance of the air gaps. Therefore, in general, it can be assumed, in accordance with the standard [24], that enlarging the gap beyond 2 cm is not justified. However, in the case of reversed heat flow there is no such strict limit. The article [48] presents data on attic overheating, i.e., downward heat flow, where the optimal cavity thickness was indicated as 5 to 7.5 cm.

## 7. Conclusions

The complex heat transfer in systems with reflective insulation (RI) poses certain challenges in properly understanding assessments and measurements of their thermal performance. The long-wave infrared emissivity coefficient, *ε*, of the reflective material’s surface is of particular importance in this case. Significant thermal resistance of RI insulation can only be expected at low values of this parameter. However, very precise and reliable measurement of this coefficient, especially at very low values, requires specialized equipment and laboratories. A visual assessment of the material surface properties may have little to do with the long-wave emissivity. This may lead to the emergence of unproven products and solutions on the building market, whose properties are far from the declared and expected results. Therefore, a verification procedure of the offered solutions is essential, as well as careful selection and easy access to reliable information in the design process.

- The validity of the proposed comparative procedure was confirmed by the good agreement between experimental measurements obtained using a guarded hot plate apparatus and calculations based on ISO standards. The proposed procedure not only enables the rough verification of the reflective properties of insulation products, which is highly relevant for construction practice, but also allows for the quantitative determination of their thermal emissivity. However, as stated earlier, the primary objective of the procedure is not to replace laboratory-grade emissivity measurements but rather to provide an accessible method for preliminary verification of material performance and manufacturer declarations.
- Five building materials marketed as possessing reflective thermal insulation properties were investigated. The results are both surprising and disappointing from the perspective of product performance. Only two of the tested materials exhibited surface layers with low long-wave emissivity (below 0.10), which is necessary for effective reflection of thermal radiation. In the remaining cases, the measured emissivity was substantially higher (above 0.59), indicating poor reflective performance.
- The surface emissivity features determined using the proposed method were consistent with observations obtained by scanning electron microscopy. The absence of aluminum particles in the outermost surface layer resulted in high thermal emissivity. Consequently, the discrepancy between the actual and declared performance of reflective insulation materials may reach several hundred percent. A sprayed aluminum layer that is too thin, or aluminum particles covered by even a very thin layer (5 micrometers) of another material, effectively eliminates reflective properties in the long-wave infrared range.
- For reflective insulation systems incorporating air layers that are 2 cm or more in thickness, the equivalent thermal conductivity of the entire assembly is significantly higher than that of conventional insulation materials of the same overall thickness. This finding questions the practical applicability of such solutions in buildings, where minimizing insulation thickness is often essential. Nevertheless, reflective insulation may be a suitable option for specialized applications with lower thermal performance requirements and a limited load-bearing capacity, such as lightweight temporary structures, warehouse buildings, or sports arena roofs.
- Claims that currently available reflective insulation materials can replace conventional insulation layers that are several times thicker are not supported by the results of this study and should be regarded as unrealistic.

## Figures and Tables

**Table 1:** Measured values of long-wave emissivity coefficient, *ε*.

Material	*ε* [−]
multilayer insulation (RI-1)	0.09
PE foam insulation (RI-2), side 1	0.68
PE foam insulation (RI-2), side 2	0.68
bubble foil (RI-3), side 1	0.59
bubble foil (RI-3), side 2	0.66
foil with large bubbles P (RI-4)	0.63
foil with large bubbles M (RI-5)	0.05
household foil, glossy side	0.05
household foil, matte side	0.06

**Table 2:** Equivalent coefficient of thermal conductivity, λ_eq_, of the system: RI + two air gaps, each 2 cm thick.

Material	λ_eq_ [W/mK]
Multilayer insulation (RI-1)	0.059
PE foam insulation (RI-2)	0.083
Bubble foil (RI-3)	0.086
Foil with large bubbles P (RI-4)	0.088
Foil with large bubbles M (RI-5)	0.062
Household foil	0.054

## Data Availability

The original contributions presented in this study are included in the article. Further inquiries can be directed to the corresponding author.

## Abbreviations

BSDs	Backscattered electron detectors
EDS	Energy dispersive spectroscopy
HFM	Heat flow meter
HFT	Heat flow transducer
RI	Reflective insulation
